# Comparative metabolic profiling of different pakchoi cultivars reveals nutritional diversity *via* widely targeted metabolomics

**DOI:** 10.1016/j.fochx.2024.101379

**Published:** 2024-04-10

**Authors:** Shiyao Dong, Siyu Fang, Jinyan Li, Wenfeng Zheng, Zhe Wang, Junlong Hu, Xiuqi Zhao, Zhiyong Liu, Hui Feng, Yun Zhang

**Affiliations:** Department of Horticulture, Shenyang Agricultural University, Shenyang, SY, China

**Keywords:** Pakchoi, Widely targeted metabolome analysis, Nutritional diversity, Differential metabolites, Phenolic acids

## Abstract

Pakchoi (*Brassica rapa* ssp. *chinensis*) is cultivated for its high nutritional value; however, the nutritional diversity of different pakchoi cultivars is rarely investigated. Herein, we performed widely targeted metabolic profiling analyses of five popular pakchois. A total of 670 metabolites were detected, which could be divided into 13 categories. The accumulation patterns of main nutritional metabolites among the five pakchois were significantly different and complementary. Moreover, the pakchoi cultivar ‘QYC’ showed quite different metabolomic profiles compared with other pakchois. The Venn diagram showed that the 75 differential metabolites were shared among the comparison groups (‘QYC’ *vs.* ‘MET’/ ‘NBC’/ ‘PPQ’/ ‘XQC’), of which 52 metabolites were upregulated in ‘QYC’. The phenolic acids had the largest variations between ‘QYC’ and the other pakchoi cultivars. These findings expand metabolomic information on different pakchoi cultivars and further provide new insights into the selection and breeding of excellent pakchoi cultivars.

## Highlights

• Each pakchoi cultivar has unique nutritional characteristics and metabolomic profiles.
• Phenolic acids and flavonoids were the main secondary metabolites in pakchoi.
• The phenolic acids had the most variation between ‘QYC’ and the other cultivars.
• Three phenolic acid metabolites were detected in pakchoi for the first time.

## 1 Introduction

Pakchoi (*Brassica rapa* ssp. *chinensis*), belonging to the Brassicaceae family, is an important leafy vegetable that is widely cultivated in Asia and the level of consumption is also rising in countries in Northern Europe and elsewhere (Chen et al., 2016). As a major popular vegetable, pakchoi is palatable and rich in various nutritional components such as vitamins, dietary fibers and minerals. The wide range of accessions in pakchoi is renowned for its high morphological diversity (Wang, Li, & Song, 2018; Zheng et al., 2015). Phenotypic heterogeneities significantly impact the metabolomic composition and nutritional characteristics of pakchois (Khan et al., 2018). The diverse nutritional and flavor characteristics of different pakchoi cultivars would attract distinct consumer preferences. Although its popularity is increasing, the nutritional quality of pak choi has not been thoroughly investigated.

As the population's pursuit of health and demand for premium vegetables increases, the focus has shifted from the basic nutrients found in primary metabolites to the functional nutrients provided by secondary metabolites (Monjotin, Amiot, Fleurentin, Morel, & Raynal, 2022; Olszewska et al., 2020). Secondary metabolites can not only be used as sunscreens by plant leaves to protect inner cells from harmful radiation but are also considered to be the major bioactive compounds in edible plants with respect to human health benefits due to their potent antioxidant capacity (Brunetti, Di Ferdinando, Fini, Pollastri, & Tattini, 2013; Del Rio et al., 2013). Pakchoi contains various secondary metabolites, including phenolic acids, flavonoids, glucosinolates, and so on (Chen et al., 2019; Heinze et al., 2018).

Phenolic acids and flavonoids, belonging to the phenolic compounds, are synthesized from phenylalanine and tyrosine *via* the shikimic acid pathway (Khanam, Oba, Yanase, & Murakami, 2012). They can act as an antioxidant, cytoprotective, vasoprotective, antiproliferative, antithrombotic and cardioprotective agent (Santos et al., 2011). To date, the main phenolic acids identified are ferulic, sinapic, caffeic and p-coumaric acids, and the main flavonoids identified are glycosylated quercetin, kaempferol, and isorhamnetin in cruciferous vegetables (Harbaum, Hubbermann, Zhu, & Schwarz, 2008; Rochfort, Imsic, Jones, Trenerry, & Tomkins, 2006). Previous studies have shown that cultivars with purple leaves accumulated more flavonoids than cultivars with green leaves, which indicated that purple pakchoi might have higher antioxidant activities than green pakchoi (Jeon, Lim, Kim, & Park, 2018; Yeo, Baek, Sathasivam, Kim, & Park, 2021). In addition, glucosinolates, mainly found in species of the *Brassicaceae* family, can be classified as aliphatic, indole, and aromatic glucosinolates based on the amino acid precursor of their side chain (Prieto, Lopez, & Simal-Gandara, 2019). Glucosinolates and their breakdown products exhibit diverse biological functions, which have been well-established in their relevance to human health (Loizzo et al., 2021; Núñez-Sánchez et al., 2022). However, although the benefits of various secondary metabolites have been addressed in the above studies, the differences in the metabolic profiles of different pakchoi cultivars are unclear.

In recent years, several studies have utilized HPLC to conduct metabolic profiling analysis on pakchoi leaves of varying colors, which revealed the correlation between leaf colour and metabolomic characteristics across different pakchoi varieties (Jeon et al., 2018; Yeo et al., 2021; Zhang et al., 2014). However, owing to the diversity of metabolites in plants, it is difficult to achieve comprehensive analysis and high throughput simultaneously. Widely targeted metabolomics is an effective method that combines the advantages of non-targeted and targeted metabolomics using the UPLC-MS/MS system (LeVatte, Keshteli, Zarei, & Wishart, 2022). This method has been widely applied in plant metabolite analysis in various species such as rice, tea and peanuts (Shi et al., 2022; Zhang et al., 2022; Zhang, Cui, Ma, Han, & Han, 2023). Although some studies have reported the compounds with health benefits in pakchoi, there is a lack of comprehensive comparative analysis of metabolites among different cultivars due to the limited diversity of cultivars.

Herein, we performed UPLC-MS/MS-based widely targeted metabolome profiling and compared the metabolites of five representative pakchoi cultivars to identify differential metabolites in different pakchoi cultivars. Differential metabolites were analyzed using multivariate statistical analysis methods such as principal component analysis (PCA), supervised orthogonal partial least squares discriminant analysis (OPLS-DA), hierarchical clustering analysis (HCA), and Kyoto Encyclopedia of Genes and Genomes (KEGG) pathway analysis. We investigated the critical corresponding differential metabolic pathways. Our findings could provide the theoretical basis for pakchoi nutritional value assessment and help identify the best cultivars according to their purpose in food processing, medicine, and plant breeding programs.

## 2 Material and methods

### 2.1 Plant materials

**Fig. 1:**
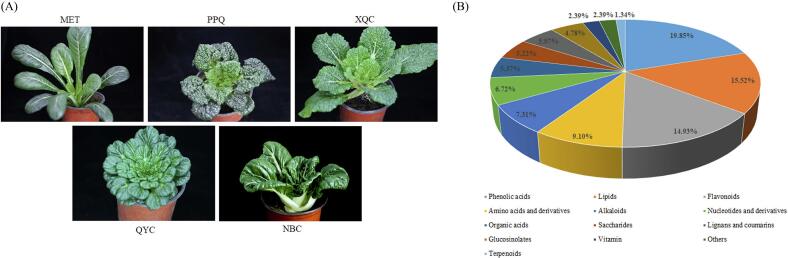
**(A)** Phenotype of five representative pakchoi cultivars ‘MET’, ‘PPQ’, ‘XQC’, ‘QYC’ and ‘NBC’. (**B)** Classification of the metabolic profiles in the five pakchoi cultivars.

Five pakchoi cultivars, ‘Maertou’ (MET), ‘Paopaoqing’ (PPQ), ‘Xiangqingcai’ (XQC), ‘Naibaicai’ (NBC) and ‘Qianyecai’ (QYC) were obtained from Liaoning province key laboratory of cruciferous vegetables (Fig. 1A). Pakchoi plants were grown at Shenyang Agricultural University in the autumn of 2022. Pakchoi leaf samples were harvested in triplicates in the shelf-life stage and immediately frozen in liquid nitrogen. Samples were stored at −80 °C until the extraction of metabolites.

### 2.2 Sample preparation for metabolite extraction

Biological samples were freeze-dried using a vacuum freeze-dryer (Scientz-100F) and then crushed for 1.5 min at 30 Hz in a mixer mill (MM 400, Retsch) with a zirconia bead. Dissolve 50 mg of lyophilized powder with 1.2 mL 70% methanol solution, vortex 30 s every 30 min for six times. Following centrifugation at 12000 rpm for 3 min, the extracts were filtrated (SCAA-104, 0.22 μm pore size; ANPEL, Shanghai, China, http://www.anpel.com.cn/) before UPLC-MS/MS analysis.

### 2.3 UPLC conditions and electrospray ionization-quadrupole-linear ion trap-tandem mass spectroscopy

The sample extracts were analyzed using a UPLC-ESI-MS/MS system (UPLC, SHIMADZU Nexera X2, https://www.shimadzu.com.cn/; MS, Applied Biosystems 4500 Q TRAP, https://www.thermofisher.cn/cn/zh/home/brands/applied-biosystems.html). The analytical conditions were as follows, UPLC: column, Agilent SB-C18 (1.8 μm, 2.1 mm*100 mm); The mobile phase has consisted of solvent A, pure water with 0.1% formic acid, and solvent B, acetonitrile with 0.1% formic acid. Sample measurements were performed with a gradient program that employed the starting conditions of 95%A, and 5%B. Within 9 min, a linear gradient to 5%A and 95%B was programmed, and a composition of 5%A and 95%B was kept for 1 min. Subsequently, a composition of 95%A and 5%B was adjusted within 1.1 min and maintained for 2.9 min. The flow velocity was 0.35 mL per minute; The column oven was set to 40 °C; The injection volume was 4 μL. The effluent was alternatively connected to an ESI-triple quadrupole-linear ion trap (QTRAP)-MS.

The ESI source operation parameters were as follows: source temperature 550 °C; ion spray voltage (IS) 5500 V (positive ion mode)/−4500 V (negative ion mode); ion source gas I (GSI), gas II(GSII), and curtain gas (CUR) were set at 50, 60, and 25 psi, respectively; the collision-activated dissociation (CAD) was high. Instrument tuning and mass calibration were performed with 10 and 100 μmol/L polypropylene glycol solutions in QQQ and LIT modes. QQQ scans were acquired as MRM experiments with collision gas (nitrogen) set to medium. DP (declustering potential) and CE (collision energy) for individual MRM transitions were done with further DP and CE optimization. A specific set of MRM transitions was monitored for each period according to the metabolites eluted within this period.

### 2.4 Multivariate statistical analysis

To study the accumulation of accession-specific metabolite, the HCA, PCA, and OPLS-DA were conducted on the metabolic data of each sample. Heatmaps with dendrograms were used to display the HCA results of both metabolites and samples. The R package heatmap was used for HCA; PCA indicates the original state metabolomic data and was used to display the variables, which was performed using GraphPad Prism v9.01 (GraphPad Software Inc., La Jolla, CA, USA). Metabolite data were unit variance scaled before HCA and PCA; An OPLS-DA model was performed using the R software package MetaboAnalystR to compare the metabolic characteristics of different rice cultivars. The metabolite data were log_2_-transformed (log_2_) to improve normality and mean centering before OPLS-DA analysis. A permutation test (200 permutations) was performed to avoid overfitting. The variable importance in the projection (VIP) ≧1 in the OPLS-DA model and the absolute Log_2_FC (fold change) ≧1 were set for screening differential metabolites. Venn diagrams were used to show the number of differential metabolites.

### 2.5 Kyoto encyclopedia of genes and genomes (KEGG) annotations and metabolic pathway analyses of differential metabolites

Identified metabolites were annotated using the KEGG Compound database (http://www.kegg.jp/kegg/compound/), and annotated metabolites were then mapped to the KEGG Pathway database (http://www.kegg.jp/kegg/pathway.html). Pathways with significantly regulated metabolites mapped were then fed into MSEA (metabolite sets enrichment analysis); their significance was determined by hypergeometric test's *p*-values.

## 3 Result

### 3.1 Overview of the metabolites in five pakchois

A total of 670 metabolites were detected in the five pakchois. The identified metabolites were divided into 13 metabolite groups, including phenolic acids (19.85%, 133), lipids (15.52%, 104), flavonoids (14.93%, 100), amino acids and derivatives (9.10%, 61), alkaloids (7.31%, 49), nucleotides and derivatives (6.72%, 45), organic acids (5.37%, 36), saccharides (5.22%, 35), lignans and coumarins (5.07%, 34), glucosinolates (4.78%, 32), vitamins (2.39%, 16), others (2.39%, 16), and terpenoids (1.34%, 9) (Fig. 1B). Thus, phenolic acids and flavonoids are the major secondary metabolites in the five pakchois. There were 649, 659, 656, 656 and 653 metabolites in ‘MET’, ‘PPQ’, ‘XQC’, ‘QYC’ and ‘NBC’, respectively.

### 3.2 Comparative analysis of main nutritional metabolites in five pakchois

To illustrate the nutritional value of different pakchois, we compared the accumulation patterns of common nutritional metabolites lipids, amino acids and derivatives, vitamins, phenolic acids, flavonoids and glucosinolates in different pakchois.

**Fig. 2:**
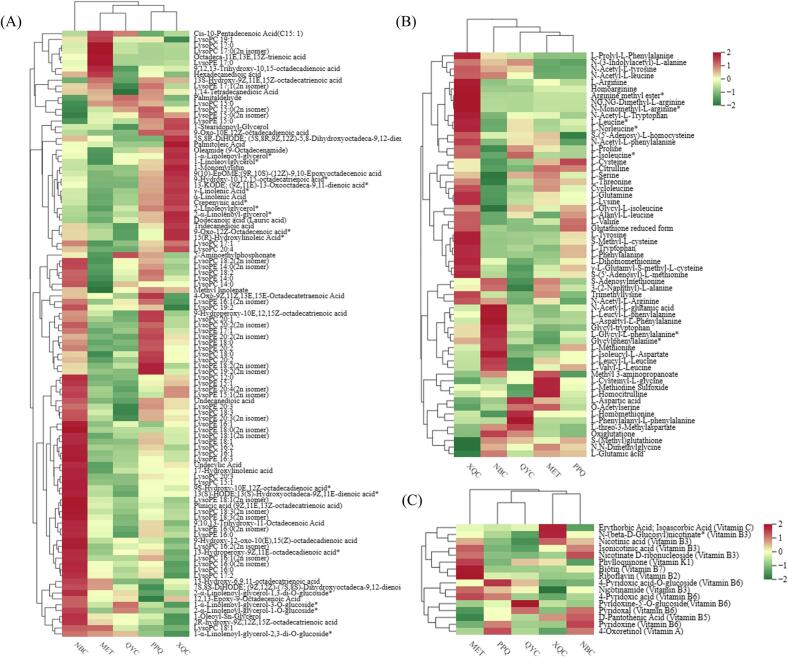
Accumulation of main nutritional primary metabolites in five pakchois. **(A)** Heat map of lipids in five pakchois; **(B)** Heat map of amino acids and derivatives in five pakchois; **(C)** Heat map of vitamins in five pakchois. The content value of each metabolite was normalized, and hierarchical cluster analysis (HCA) was performed. Each pakchoi species is represented by a column, and each metabolite is displayed in a row. Red shows relatively high metabolite abundance, while green indicates relatively low abundance. (For interpretation of the references to colour in this figure legend, the reader is referred to the web version of this article.)

Lipid metabolites were widely detected in pakchois, including 30 lysophosphatidyl cholines (LPCs), 25 lysophosphatidyl ethanolamines (LPEs), 38 free fatty acids (FFAs) and 11 glycerol esters (Table S1). The relative content of total lipid metabolites was highest in ‘NBC’ and lowest in ‘QYC’ (Fig. 2A). ‘NBC’ was rich in most lysoPC metabolites, including lysoPC16:0, lysoPC16:1, lysoPC18:1 and lysoPC18:3, while the relative content of lysoPC18:3, lysoPC19:2 and lysoPC20: 2 were higher in ‘PPQ’. In addition, ‘XQC’ contained more FFA metabolites such as α-linolenic acid, γ-linolenic acid and 15(*R*)-hydroxylinoleic acid (Fig. 2A).

16 proteinogenic and 45 non-proteinogenic amino acids were confirmed to exist in different pakchoi cultivars (Table S2). The relative content of amino acid metabolites in ‘XQC’ was the most abundant (Fig. 2B). The eight essential amino acids were detected in pakchoi. Among them, only the relative content of methionine was highest in ‘NBC’ while the others were highest in ‘XQC’. The relative content of most non-proteinogenic amino acids was most elevated in ‘XQC’, followed by ‘NBC’ (Fig. 2B).

Vitamin A, B, C, and K were enriched in various cultivars of pakchoi, particularly vitamin B, including vitamin B_2_, B_3_, B_5_, B_6_, B_7_ and derivatives (Table S3). The metabolite contents of vitamins varied in different pakchoi cultivars. The relative content of N-(beta-D-Glucosyl) nicotinate (vitamin B_3_), 4-pyridoxic acid-O-glucoside (vitamin B_6_) and pyridoxine-5’-O-glucoside (vitamin B_6_) were highest in ‘XQC’, ‘PPQ’ and ‘QYC’, respectively. The relative content of biotin (vitamin B_7_) and riboflavin (vitamin B_2_) were significantly higher in ‘MET’ than others. The ‘PPQ’ and ‘NBC’ exhibited a relatively high 4-oxoretinol (vitamin A) content, while ‘XQC’ had the highest content of isoascorbic acid (vitamin C). Additionally, phylloquinone (vitamin K_1_) levels were comparatively higher in ‘MET’, ‘XQC’, and ‘QYC’ (Fig. 2C).

**Fig. 3:**
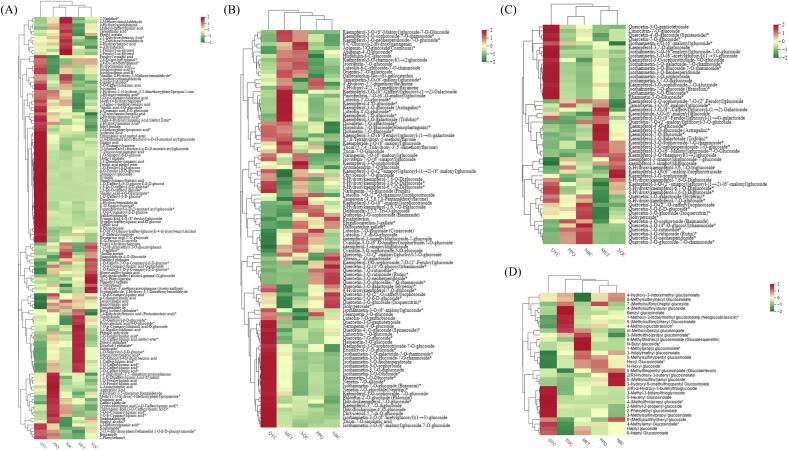
Accumulation of main nutritional secondary metabolites in the five pakchoi cultivars. **(A)** Heat map of phenolic acids in the five pakchois; **(B)** Heat map of flavonoids in the five pakchois; **(C)** Heat map of flavonols in the five pakchois; **(D)** Heat map of glucosinolates in the five pakchois. The content value of each metabolite was normalized, and hierarchical cluster analysis (HCA) was performed. Each pakchoi species is represented by a column, and each metabolite is displayed in a row. Red shows relatively high metabolite abundance, while green indicates relatively low abundance. (For interpretation of the references to colour in this figure legend, the reader is referred to the web version of this article.)

Phenolic acid metabolites mainly existing as bound phenolics were the most abundant among the five pakchoi cultivars (Table S4). The relative content of phenolic acid metabolites was highest in ‘QYC’ and lowest in ‘XQC’ (Fig. 3A). In particular, the relative contents of isoferulic acid, sinapic acid, caffeic acid, isochlorogenic acid B/C and derivatives in ‘QYC’ were higher than those in the other cultivars. Furthermore, rosmarinic acid-3’-O-glucosidesyringin, syringin and coniferin were identified in pakchoi for the first time. Cinnamic acid and the isomers of chlorogenic acid (cryptochlorogenic acid, neochlorogenic acid and cynarin) displayed higher levels in ‘PPQ’ than in the other cultivars. Protocatechuic acid levels were higher in ‘MET’ than others but absent in ‘XQC’ (Fig. 3A).

As shown in Table S5, the flavonoid metabolites in five pakchois were different and complementary, including 53 flavonols, 27 flavones, 8 flavanones, 4 flavanols, 3 flavanonols, 3 anthocyanidins and 2 chalcones. The relative content of flavonoid metabolites was higher in ‘QYC’ and ‘MET’, and lower in ‘NBC’ and ‘PPQ’ (Fig. 3B). Among them, the flavonols accounted for 53% of the detected flavonoids and mainly comprised three significant kinds: glycosylated kaempferol, glycosylated isorhamnetin, glycosylated quercetin and derivatives. Our study found that the relative contents of the most glycosylated kaempferol in ‘MET’ and ‘XQC’ were higher than those in the other cultivars, glycosylated isorhamnetin accumulated significantly in ‘QYC’, and glycosylated quercetin accumulated highly in ‘NBC’. However, flavonol metabolites in ‘PPQ’ showed no obvious accumulation pattern (Fig. 3C).

Furthermore, glucosinolate metabolites, a critical secondary metabolite in cruciferous vegetables, mainly included aliphatic and aromatic glucosinolates in five pakchois (Table S6). The relative content of glucosinolate metabolites was highest in ‘QYC’ and lowest in ‘NBC’. Glucosinolate metabolites exhibited prominent accumulation characteristics in ‘QYC’, ‘XQC’, ‘NBC’ and ‘MET’ apart from ‘PPQ’ (Fig. 3D).

### 3.3 Multivariate statistical analysis

**Fig. 4:**
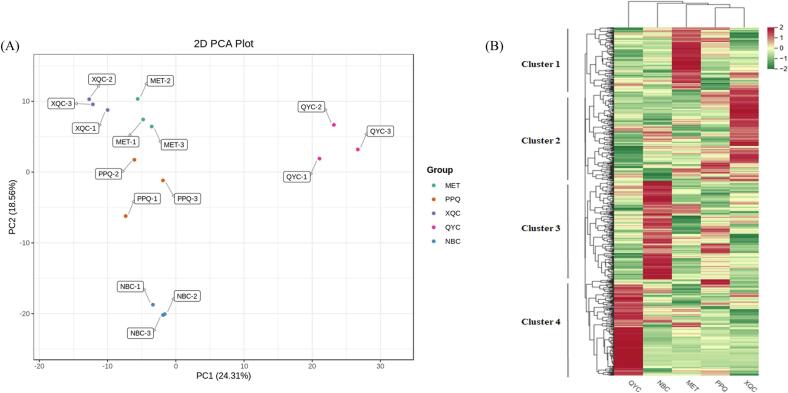
Principal component analysis (PCA) and hierarchical cluster analysis (HCA) of the metabolites in five pakchoi cultivars. (**A**) PCA score plot; (**B)** HCA map of all metabolites. Each sample is represented by a column, and each metabolite is represented by a row. The abundance of each metabolite is represented by a bar with a specific colour. The upregulated and downregulated metabolites are indicated by different shades of red and green, respectively. (For interpretation of the references to colour in this figure legend, the reader is referred to the web version of this article.)

To further explore the diversity of metabolites among the five pakchois, we analyzed the metabolic profile using principal component analysis (PCA), and hierarchical cluster analysis (HCA). The PCA reflects the overall metabolic differences among each group and the degree of variation between samples within the group. Principal component 1(PC1) and Principal component 2 (PC2) contributed to 24.31% and 18.56% of the variance, respectively. Furthermore, ‘MET’, ‘PPQ’, ‘NBC’ and ‘XQC’ clustered together, whereas ‘QYC’ was far from the others in PC1. Meanwhile, ‘NBC’ was separated from other cultivars in PC2 (Fig. 4A).

HCA identified four major clusters based on the variations in the relative quantity of the metabolites. Metabolites involved in cluster 1 accumulated at the highest levels in ‘MET’, while cluster 2 had their highest levels in ‘XQC’, cluster 3 had their highest levels in ‘NBC’, and cluster 4 were the highest in ‘QYC’. There was no apparent cluster of metabolites in ‘PPQ’. The results of horizontal cluster analysis also indicated that ‘QYC’ and the other four cultivars belong to two distinct branches (Fig. 4B). Three biological replicates were performed in each group, demonstrating sufficient repeatability among the replicates and high data reliability. Overall, these results indicated that the metabolic profiles in the five samples were significantly different and the metabolite accumulation pattern in ‘QYC’ was quite different from those in the other cultivars.

### 3.4 Identification of differential metabolites between ‘QYC’ and the other cultivars

Based on the K-means cluster analysis, we found two different change tendencies of the 380 differential metabolites among the five pakchoi cultivars. Among them, 163 metabolites showed a higher content in ‘QYC’ than in the others (Fig. S1A), and 217 metabolites had a lower content (Fig. S1B). Upregulated metabolites from subclass 1 in ‘QYC’ mainly included 54 phenolic acids and 32 flavonoids (Table S7). Downregulated metabolites from subclass 2 in ‘QYC’ mainly included 20 lipids. The regularity of other types of compounds was not obvious (Table S8).

**Fig. 5:**
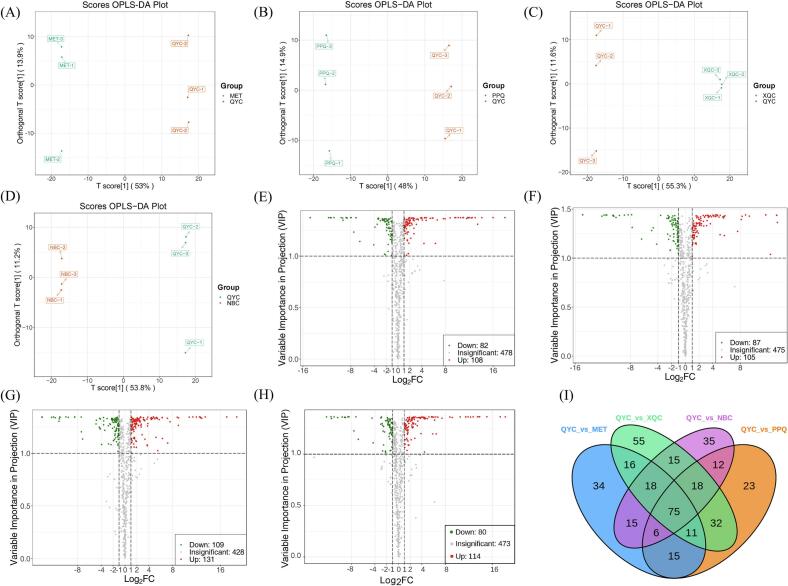
Differential metabolites and Venn diagram analysis of ‘QYC’ compared with ‘MET’/ ‘PPQ’/ ‘XQC’/ ‘NBC’. Score plots generated from orthogonal partial least squares discriminant analysis (OPLS-DA) in ‘QYC’ *vs.* ‘MET’ **(A)**, ‘QYC’ *vs.* ‘PPQ’ **(B)**, ‘QYC’ *vs.* ‘XQC’ **(C)** and ‘QYC’ *vs.* ‘NBC’ **(D)**; Volcano plots showing the levels of the differential metabolites in ‘QYC’ *vs.* ‘MET’ **(E)**, ‘QYC’ *vs.* ‘PPQ’ **(F)**, ‘QYC’ *vs.* ‘XQC’ **(G)**, and ‘QYC’ *vs.* ‘NBC’ **(H); (I)** Venn diagram illustrating the overlapping and specific differential metabolites for the four comparisons (‘QYC’ *vs.* ‘MET’, ‘QYC’ *vs.* ‘PPQ’, ‘QYC’ *vs.* ‘XQC’**,** ‘QYC’ *vs.* ‘NBC’).

We used the supervised method, OPLS-DA, to screen the variables responsible for the differences among the five groups. All metabolites of pakchois were evaluated using pairwise comparison based on the OPLS-DA model to determine the differences between ‘QYC’ and ‘MET’ (R^2^X = 0.669, R^2^Y = 1, Q^2^ = 0.95), between ‘QYC’ and ‘PPQ’ (R^2^X = 0.63, R^2^Y = 0.999, Q^2^ = 0.929), between ‘QYC’ and ‘XQC’ (R^2^X = 0.668, R^2^Y = 1, Q^2^ = 0.955) and between ‘QYC’ and ‘NBC’ (R^2^X = 0.651, R^2^Y = 1, Q^2^ = 0.956) (Fig. S2). The Q^2^ values of all comparisons exceeded 0.9, demonstrating that these models were stable. The OPLS-DA score plots showed that ‘QYC’ was well-separated from the other four cultivars, which could be used to further screen out the differentially accumulated metabolites (DAMs) using VIP analysis (Fig. 5A-D).

To examine the most meaningful metabolic changes between ‘QYC’ and the other cultivars, differential metabolite screening based on the fold-change and the variable importance in the project (VIP) scores was performed among all metabolites annotated. The results are visually illustrated using Volcano plots, with a criterion of fold-change score ≥ 2 or ≤ 0.5 and VI*P* value≥1. In summary, there were 190 significantly different metabolites (108 upregulated and 82 downregulated) between ‘QYC’ and ‘MET’ (Fig. 5E), 192 (105 upregulated and 87 downregulated) between ‘QYC’ and ‘PPQ’ (Fig. 5F), 240 (131 upregulated and 109 downregulated) between ‘QYC’ and ‘XQC’ (Fig. 5G), and 194 (114 upregulated and 80 downregulated) between ‘QYC’ and ‘NBC’ (Fig. 5H). The results showed the highest number of DAMs between ‘QYC’ and ‘XQC’. After taking the intersection of each comparison group in a Venn diagram, 75 metabolites were shared among the comparison groups (‘QYC'*vs.* ‘MET’/ ‘NBC’/ ‘PPQ’/ ‘XQC’) (Fig. 5I). Besides, OPLS-DA analysis was conducted on five cultivars of pakchois. The results demonstrated obvious separation between different groups (Fig. S4). By applying VIP value≥1 and P value<0.05 as criteria, a total of 192 differential metabolites were identified in all cultivars, among which phenolic acids and lipids being the predominant classes (Table S9).

### 3.5 KEGG annotation and enrichment analysis of differential metabolites

**Fig. 6:**
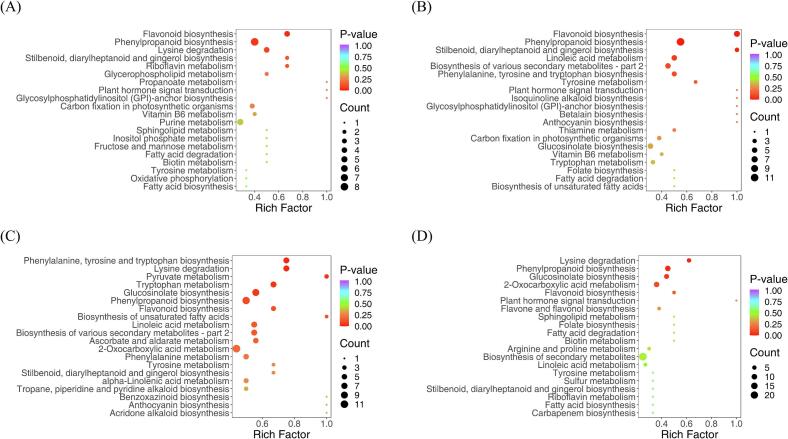
Kyoto Encyclopedia of Genes and Genomes (KEGG) annotations and enrichment results of the differentially accumulated in the pairwise comparison between ‘QYC’ *vs.* ‘MET’ **(A)**; ‘QYC’ *vs.* ‘PPQ’ **(B)**; ‘QYC’ *vs.* ‘XQC’ **(C)**; and ‘QYC’ *vs.* ‘NBC’ **(D)**.

In this study, we annotated and enriched the differential metabolites for each comparison group and divided them into different KEGG pathways. The differential metabolites in the ‘QYC’ *vs.* ‘MET’/ ‘PPQ’/ ‘XQC’/ ‘NBC’ groups according to the KEGG database were enriched in 50, 60, 64, and 46 pathways, and the major pathways are illustrated in bubble plots (Fig. 6A-D). Metabolic pathways related to “flavonoid biosynthesis” and “phenylpropanoid biosynthesis” were significantly enriched (*p*-value <0.05) in ‘QYC’ *vs.* ‘MET’ (Fig. 6A). In ‘QYC’ *vs.* ‘PPQ’, metabolic pathways related to “phenylpropanoid biosynthesis”, “flavonoid biosynthesis”, and “stilbenoid, diarylheptanoid and gingerol biosynthesis” were significantly enriched (Fig. 6B). In ‘QYC’ *vs.* ‘XQC’, “lysine degradation”, “phenylalanine, tyrosine and tryptophan biosynthesis”, “pyruvate metabolism”, and “tryptophan metabolism” were the significantly enriched metabolic pathways (p-value <0.05) (Fig. 6C); when comparing ‘QYC’ *vs.* ‘NBC’, “phenylpropanoid biosynthesis” and “lysine degradation” was the significantly enriched metabolic pathways (p-value <0.05) (Fig. 6D). The differential metabolites in ‘QYC’ compared with the others were significantly enriched in phenylpropanoid biosynthesis.

### 3.6 Key significantly differential metabolites between ‘QYC’ and the other cultivars

To explore the key differential metabolites of ‘QYC’, a Venn diagram was produced to illustrate the relationship between joint differential metabolites among ‘QYC’ *vs.* ‘MET’/ ‘PPQ’/ ‘XQC’/ ‘NBC’. In the four pairwise comparisons, 75 overlapping differential metabolites of which 52 were upregulated in ‘QYC’, including 32 phenolic acids, 7 flavonoids, 5 glucosinolates, 3 lignans and coumarins, 3 organic acids, 1 amino acid and derivatives and 1 saccharide (Table 1). Phenolic acids and flavonoids accounted for 61.54% and 13.46% of the key differential metabolites, respectively.

**Table 1:** Fifty-two key significantly upregulated metabolites between ‘QYC’ and the other pakchoi cultivars.

Class	Compounds	Log_2_FC (‘QYC’ *vs.* ‘MET’)	Log_2_FC (‘QYC’ *vs.* ‘PPQ’)	Log_2_FC (‘QYC’ *vs.* ‘XQC’)	Log_2_FC (‘QYC’ *vs.* ‘NBC’)
Phenolic acids	1-O-Caffeoyl-β-d-glucose	5.78	5.63	5.25	5.66
	Vanillic Acid-4-O-Glucuronide	5.75	5.62	5.33	5.66
	(3-Mustardacyl)-fructosyl-α-D-(6-mustard acyl) glucoside	2.00	1.88	1.92	3.45
	4-O-(6’-O-Glucosylcaffeoylglucosyl)-4-hydroxybenzyl alcohol	1.08	2.22	1.36	1.86
	Syringin	3.97	4.36	4.41	5.64
	Coniferin	5.88	5.76	5.33	5.83
	1-O-Sinapoyl-β-d-glucose	3.82	3.31	2.66	4.48
	3,6’-Disinapoylsucrose	1.95	1.94	2.11	3.57
	Sinapic acid	2.15	1.67	2.52	2.92
	6’-O-Sinapoylsucrose	2.63	2.80	2.13	2.26
	Furanofructosyl-α-D-(3-mustard acyl) glucoside	2.71	2.79	2.12	2.39
	6-O-Feruloyl-β-d-glucose	3.99	4.58	4.85	4.24
	1-O-Feruloyl-β-d-glucose	3.95	5.57	4.74	4.01
	1,6-Di-O-caffeoyl-β-d-glucose	7.63	9.30	7.57	18.21
	3,6-Di-O-caffeoyl glucose	7.92	8.25	7.10	7.42
	Chlorogenic acid methyl ester	3.07	1.64	4.33	2.72
	Gallacetophenone	1.86	1.56	1.71	1.54
	5′-Glucosyloxyjasmanic acid	3.88	3.46	2.61	5.19
	Syringoylcaffeoylquinic acid-d-glucose	5.40	4.01	6.25	3.85
	β-Oxoacteoside	2.00	3.19	1.74	3.74
	Disinapoyl glucoside	4.45	4.14	4.41	6.37
	1-O-p-Coumaroyl-β-d-glucose	6.59	2.64	3.30	3.17
	Sinapoylsinapoyltartaric acid	4.53	5.08	5.73	6.17
	Ferulic acid methyl ester	3.32	4.69	5.24	4.26
	3,4-Dimethoxycinnamic acid	3.42	4.64	5.23	4.41
	1-O-Caffeoyl-(6-O-glucosyl)-β-d-glucose	4.10	4.90	3.69	4.62
	6’-O-Feruloyl-D-sucrose	1.81	3.10	2.86	1.47
	4-Hydroxybenzaldehyde	1.76	1.73	1.42	1.34
	Methyl sinapate	2.69	3.37	3.20	4.44
	Syringic acid-4-O-(6′-feruloyl) glucoside	14.10	4.42	4.44	3.74
	Rosmarinic acid-3’-O-glucoside	2.64	5.68	13.47	2.44
	(3,4-Dimustardacyl)-fructosyl-α-D-(6-mustard acyl) glucoside	11.88	2.04	2.54	11.88
Flavonoids	Kaempferol-3,7-O-diglucoside	1.03	2.03	1.26	1.83
	Naringenin-4’-O-glucoside	1.95	1.58	2.55	1.19
	Isorhamnetin-3-O-neohesperidoside	1.18	2.11	2.25	2.48
	Tricin-7-O-saccharic acid	2.44	3.62	3.81	2.84
	Isorhamnetin-3-O-(6′-malonyl) glucoside-7-O-glucoside	1.42	1.64	1.96	1.63
	Dihydrocharcone-4’-O-glucoside	1.72	1.67	1.52	2.26
	Isorhamnetin-3-O-glucoside-7-O-rhamnoside	1.08	1.94	2.36	2.28
Glucosinolates	2(*R*)-2-Hydroxy-3-butenylthioglycoside	1.48	2.09	18.75	2.30
	2-Phenylethyl glucosinolate	1.54	1.08	1.51	2.23
	3-Methyl-3-butenylthioglycoside	1.50	2.16	8.96	2.43
	5-Hexenyl Glucosinolate	3.98	5.43	17.07	6.72
	4-(Methylthio)propyl glucoside	8.76	8.76	8.76	8.76
Lignans and Coumarins	Lariciresinol-4’-O-glucoside	2.26	3.33	3.10	2.54
	Secoisolariciresinol 4-O-glucoside	2.06	2.60	2.63	2.55
	Medioresinol-4,4′-di-O-glucoside	1.55	1.45	1.21	1.43
Organic acids	3-Methylmalic acid	1.93	1.01	2.26	1.39
	2-Hydroxyglutaric Acid	2.29	1.23	2.39	1.30
	Glutaric acid	4.73	2.24	5.50	4.92
Saccharides	2-Dehydro-3-deoxy-L-arabinonate	1.89	1.16	3.07	1.57
Amino acids and derivatives	L-Phenylalanyl-*L*-phenylalanine	15.98	7.60	4.62	6.04

Based on the KEGG annotation and enrichment maps, the metabolic pathways of the most relevant overlapped differential metabolites were filtered out (Fig. S3). Some phenolic acid metabolites, including syringin, coniferin, 1-O-Sinapoyl-β-d-glucose and sinapic acid were upregulated. Besides, seven flavonoid metabolites, including four flavonols (kaempferol-3,7-O-diglucoside, isorhamnetin-3-O-neohesperidoside, isorhamnetin-3-O-glucoside-7-O-glucoside, and isorhamnetin-3-O-glucoside-7-O-rhamnoside), one flavonoid (tricin-7-O-saccharic acid), one flavanone (naringenin-4’-O-glucoside) and one chalcone (dihydrochalcone-4’-O-glucoside), exhibited higher relative content in ‘QYC’. Furthermore, five glucosinolates (2(*R*)-2-Hydroxy-3-butenylthioglycoside, 2-Phenylethyl glucosinolate, 3-Methyl-3-butenylthioglycoside, 5-Hexenyl glucosinolate and 4-(Methylthio)propyl glucoside) were upregulated in ‘QYC’. Among them, 4-(Methylthio)propyl glucoside was only detected in ‘QYC’.

## 4 Discussion

### 4.1 Metabolites identified in the five pakchoi cultivars

Pakchoi is becoming increasingly popular because of its numerous cultivars and abundant nutritional benefits. Metabolomics analysis plays a critical role in revealing nutritional metabolites among different plant cultivars (Zhou et al., 2020). Previous studies identified 9 phenylpropanoid-derived compounds in green and purple pakchoi and 53 primary metabolites in white, pale green, and green pakchoi using HPLC (Jeon et al., 2018; Yeo et al., 2021). In the present study, we performed a widely targeted metabolome analysis of five representative pakchoi cultivars. We detected 670 metabolites, including phenolic acids, lipids, flavonoids, amino acids and derivatives, glucosinolates, vitamins, *etc.* In comparison to previous studies (Jeon et al., 2018; Yeo et al., 2021; Zhou et al., 2020), our investigation revealed a higher number and more abundant types of metabolites among the five pakchoi cultivars.

Among the identified metabolites, the major secondary metabolites were phenolic acids and flavonoids, which can scavenge free radicals, superoxide free radicals, and hydroxyl free radicals and exhibit high antioxidant properties (Kim, Jeong, & Lee, 2003). Significantly, glycosylated rosmarinic acid, syringin and coniferin were identified in pakchois for the first time. As a natural antioxidant, rosmarinic acid promotes the contraction of the gastrointestinal tract to help digestion, stimulates liver cell regeneration, and reduces the risk of liver damage (Adomako-Bonsu, Chan, Pratten, & Fry, 2017); syringin has anti-cancer, anti-obesity, and anti-inflammatory effects, and can be used in the treatment of ischemic stroke (Aventurado, Castillo, & Vasquez, 2022; Dong et al., 2021; Liu, Zhu, Tong, & Tan, 2022).

Flavonol, which plays an active role in preventing and treating cardiovascular and cerebrovascular diseases and reducing the brittleness of blood vessels and the contents of blood lipids and cholesterol, is the most ubiquitous flavonoid sub-class (Kubina et al., 2022; Rauf, Imran, Patel, Muzaffar, & Bawazeer, 2017). Herein, we detected that flavonol metabolites accounted for 53% of the total flavonoid metabolites. Flavonols accounted for 53% of the total flavonoid metabolites and mainly included glycosylated quercetin, kaempferol, isorhamnetin and derivatives. The previous study on metabolite analysis of 12 cruciferous vegetables revealed quercetin aglycone was detected in all tested vegetables, while kaempferol aglycone was not detected. Isorhamnetin aglycone was only found in choysum and pakchoi (Li et al., 2018); however, the presence of glycosylated quercetin, kaempferol, and isorhamnetin was observed in all five pakchoi cultivars, which diverges from the findings reported. Our results provided reference values for the isolation and identification of functional compounds of pakchois.

### 4.2 Differential metabolites between ‘QYC’ and other cultivars

Each cruciferous vegetable has its unique accumulation profile of phenolic metabolites (Li et al., 2018). Previous studies have also reported significant variation in the accumulation and content of phenolic compounds in different pakchoi cultivars (Lee et al., 2018; Park, Yeo, Park, Kim, & Park, 2019). In this study, HCA demonstrated that phenolic acid compositions and relative contents in the different pakchoi cultivars showed marked variation similar to previous studies. Most phenolic acid metabolites were relatively abundant in ‘QYC’. For the flavonoid metabolites, we found that the relative content of glycosylated kaempferol in ‘MET’ and ‘XQC’ was higher than in the others, glycosylated isorhamnetin showed a relatively higher content in ‘QYC’, and glycosylated quercetin was highly accumulated in ‘NBC’. These results indicated that the phenolic metabolites might vary among the different pakchoi cultivars, leading to their different antioxidant capacity.

In addition, the result of PCA demonstrated that the metabolite accumulation pattern in ‘QYC’ was quite different from that in other cultivars. A total of 380 differential metabolites were identified in the four comparison groups (‘QYC’ *vs.* ‘MET’ group, ‘QYC’ *vs.* ‘PPQ’ group, ‘QYC’ *vs.* ‘XQC’ group, and ‘QYC’ *vs.* ‘NBC’ group). Among the four pairwise comparisons, 52 overlapping upregulated differential metabolites in ‘QYC’ were the key metabolites, of which phenolic acid metabolites accounted for 61.54%. In particular, sinapic acid, which has been reported as a major phenolic compound in pakchoi cultivars, was present at significantly higher levels in the free and most bound fractions of ‘QYC’ than those in the other pakchois (Nguyen, Stewart, Ioannou, & Allais, 2021). Furthermore, rosmarinic acid has stronger antioxidant activity than caffeic acid and chlorogenic acid, which were highly accumulated in ‘QYC’ (Ribeiro-Santos et al., 2015). Some of the highly accumulated flavonoids in ‘QYC’ possess antioxidant bioactivities and other health benefits, including kaempferol-3,7-O-diglucoside, naringenin-4’-O-glucoside, tricin-7-O-saccharic acid, and some isorhamnetins and their aglycons (Du et al., 2021; Duarte-Almeida, Negri, Salatino, de Carvalho, & Lajolo, 2007; Wang et al., 2018). The KEGG metabolic pathway analysis showed that the “phenylpropanoid biosynthesis” metabolic pathway was the differential metabolic pathway in common between the ‘QYC’ and the other four cultivars. The phenylpropane pathway is a general pathway for synthesizing polyphenols, and all metabolites containing the phenylpropane skeleton, such as phenolic acids and flavonoids, are directly or indirectly synthesized by this pathway (Huang et al., 2022). In conclusion, identifying these differential metabolites will contribute to the nutritional evaluation of different cultivars and promote the selection and breeding of excellent pakchoi cultivars.

## 5 Conclusions

In this study, the metabolic profiles of five pakchoi cultivars were systematically evaluated to investigate the differences in metabolites based on the widely targeted metabolomics approach. A total of 670 metabolites were detected and were divided into 13 categories. The accumulation patterns of main nutritional metabolites varied with different pakchoi cultivars. ‘QYC’ had a significantly different metabolomic profile than the other cultivars, mainly reflected in the phenolic acid metabolites. The present work will contribute substantially to our knowledge of metabolite compositions of the five pakchoi cultivars and help breeders screen target cultivars with functional and nutritional properties through comparative evaluation.

## CRediT authorship contribution statement

Shiyao Dong*: Investigation, writing-original draft and data analysis. Siyu Fang*: Designed and performed the experiments. *These authors contributed to the work equally and should be regarded as co-first authors. **Jinyan Li, Wenfeng Zheng, Zhe Wang, Junlong Hu and Xiuqi Zhao:** Investigation and collected the literature. **Zhiyong Liu:** Conceptualization and writing-review. **Hui Feng:** Conceived and designed the experimental work. **Yun Zhang**: Funding acquisition, writing-review and supervision. All authors read and approved the final manuscript.

## Declaration of competing interest

The authors declare that they have no competing financial interests or personal relationships that could have appeared to influence the work reported in this paper.

## Data Availability

Data will be made available on request.

## Appendix A Supplementary data

Table S1. List of lipid metabolites identified in five pakchoi cultivars Image 1

Table S2. List of amino acid and derivative metabolites identified in five pakchoi cultivars Image 2

Table S3. List of vitamin metabolites identified in five pakchoi cultivars Image 3

Table S4. List of phenolic acid metabolites identified in five pakchoi cultivars Image 4

Table S5. List of flavonoid metabolites identified in five pakchoi cultivars Image 5

Table S6. List of glucosinolate metabolites identified in five pakchoi cultivars Image 6

Table S7. List of K-Means clustering information for differential metabolites (Subclass 1) Image 7

Table S8. List of K-Means clustering information for differential metabolites (Subclass 2) Image 8

Table S9. The key differential metabolites among the five pakchoi cultivars Image 9

Supplementary material 10 Image 10
