# Supplementary material for: Comparative metabolic profiling of different pakchoi cultivars reveals nutritional diversity via widely targeted metabolomics
Source: Food Chem X. 2024 Apr 10;22:101379. doi: 10.1016/j.fochx.2024.101379 (PMC11031806; doi:10.1016/j.fochx.2024.101379)
Supplement: Supplementary file 1 — Table S1. List of lipid metabolites identified in five pakchoi cultivars [file mmc1.docx]

**
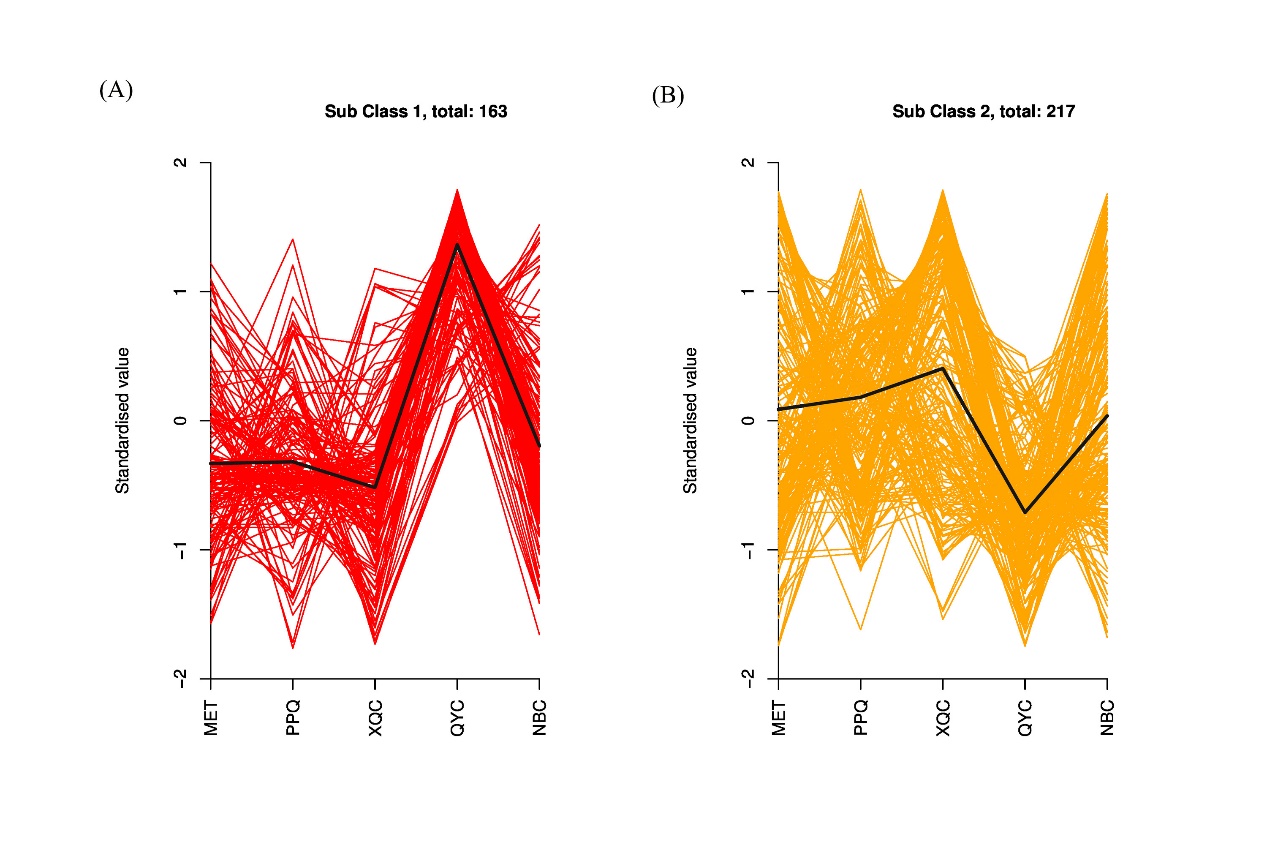
**

**Fig. S1** K-means clusters of the expression profiles of five pakchois. **(A)** Up-regulated DAMs in ‘QYC’; **(B)** Down-regulated DAMs in ‘QYC’. The y-axis represents the normalized metabolite content and the x-axis represents the different samples.


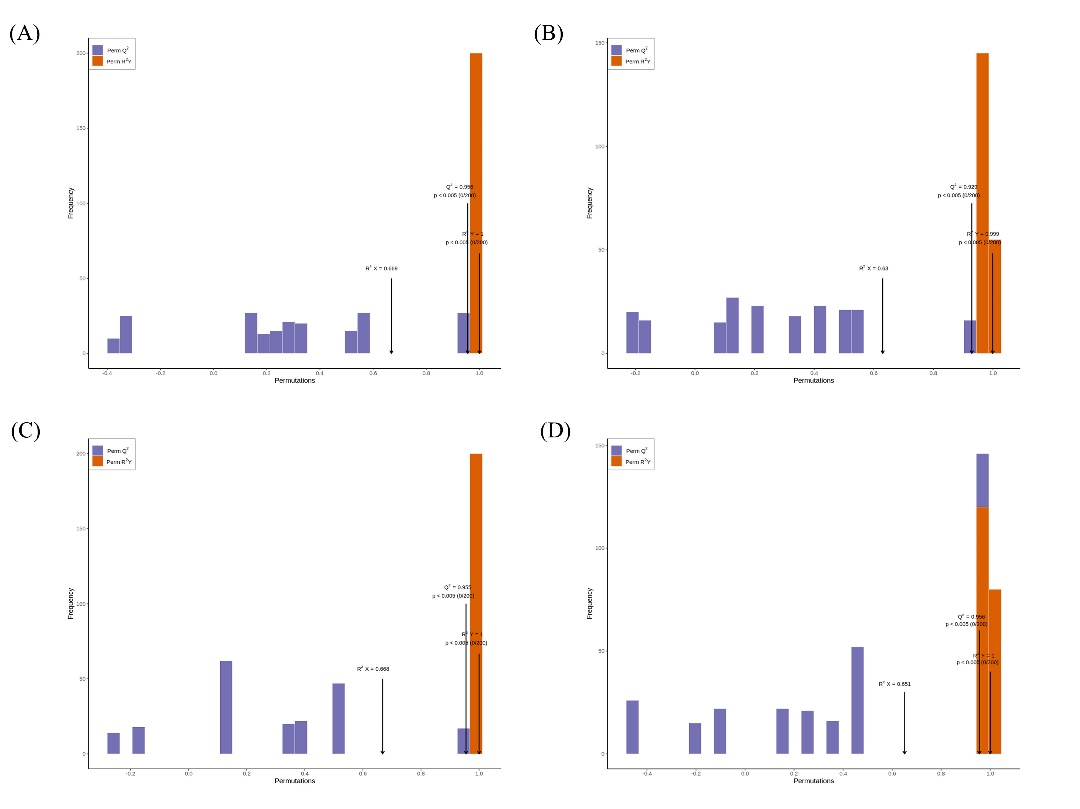


**Fig. S2** OPLS-DA model verification diagram. **(A)** ‘QYC’ and ‘MET’; **(B)** ‘QYC’ and ‘PPQ’; **(C)** ‘QYC’ and ‘XQC’; **(D)** ‘QYC’ and ‘NBC’.


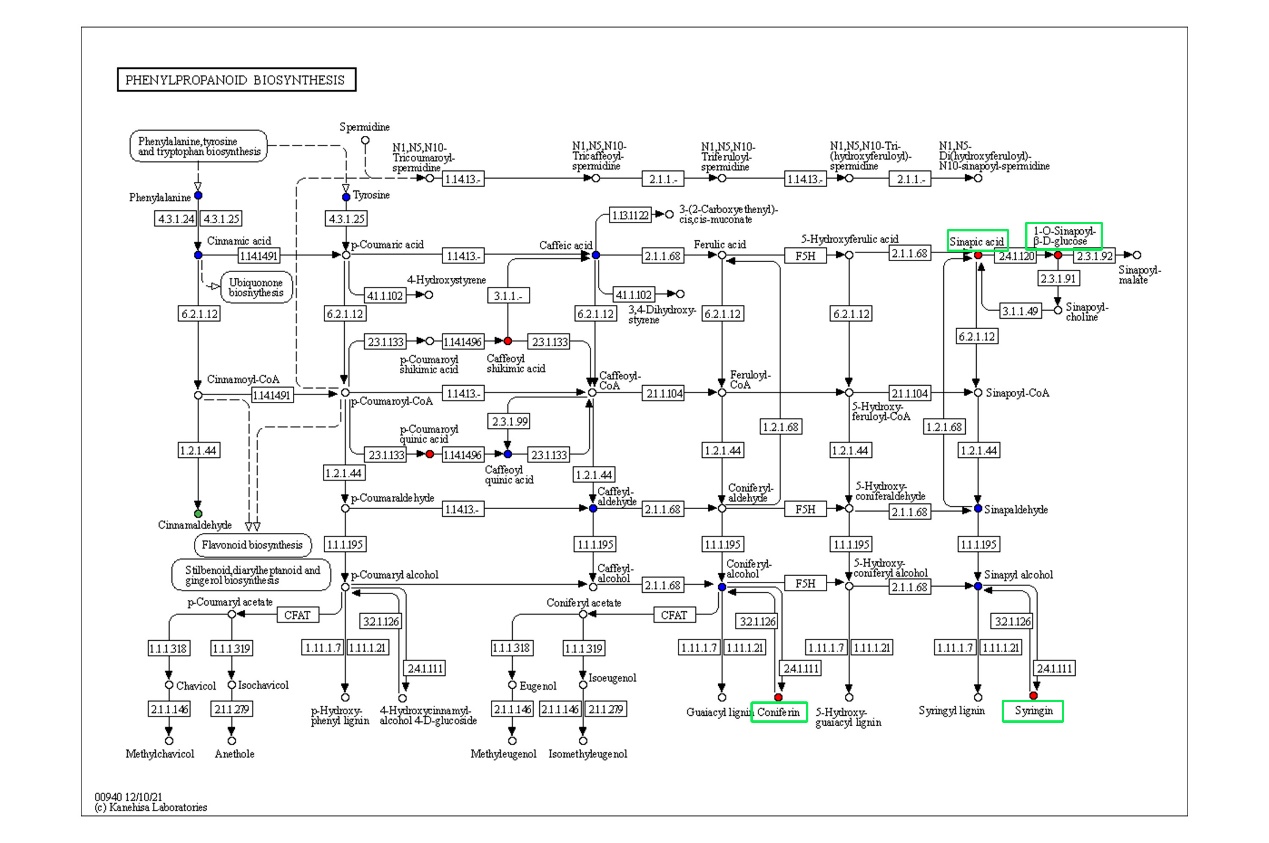


**Fig. S3** The KEGG pathways of “phenylpropanoid biosynthesis”. Green boxes represented metabolites on “phenylpropanoid biosynthesis” metabolic pathway that overlap with fifty-two key significantly upregulated metabolites between ‘QYC’ and the other pakchoi cultivars


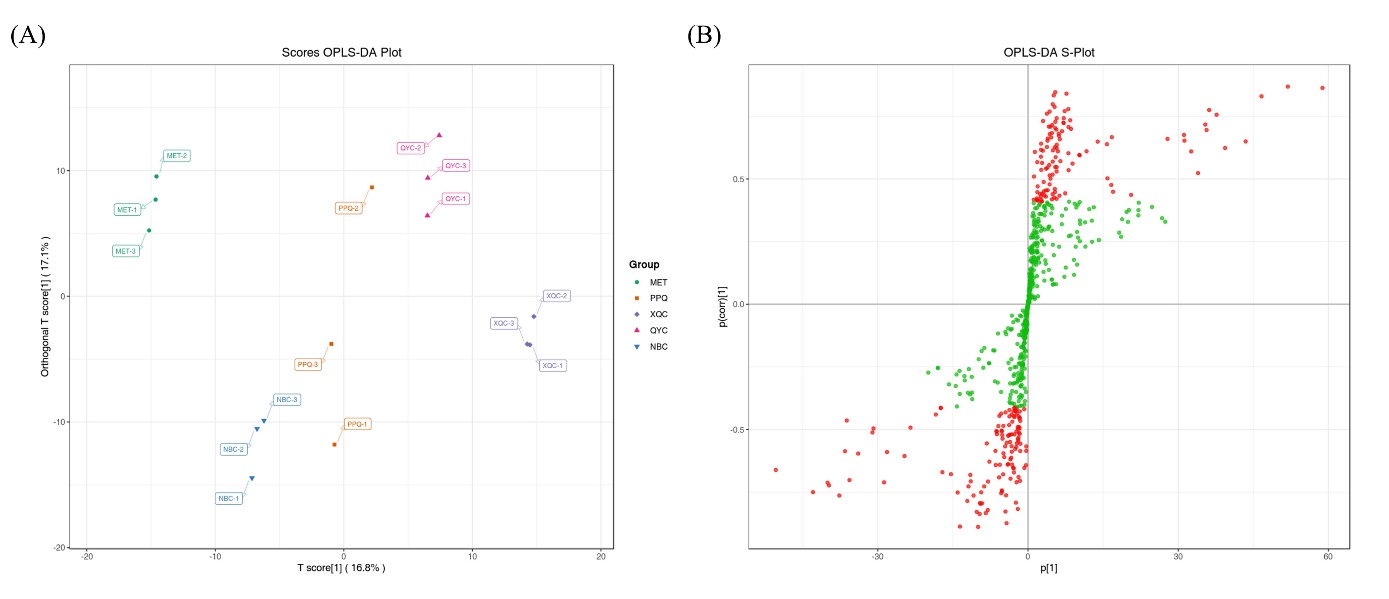


**Fig. S4 (A)** OPLS-DA score plot of five pakchoi cultivars. **(B)** OPLS-DA S-Plot of five pakchoi cultivars. The red points in the S-plot diagram represented the metabolites VIP value≥1 and the green points represented the metabolites VIP value≤1.
